# Supplementary figures and images for: Effects of macroalgae loss in an Antarctic marine food web: applying extinction thresholds to food web studies
Source: PeerJ. 2018 Sep 12;6:e5531. doi: 10.7717/peerj.5531 (PMC6139014; doi:10.7717/peerj.5531)

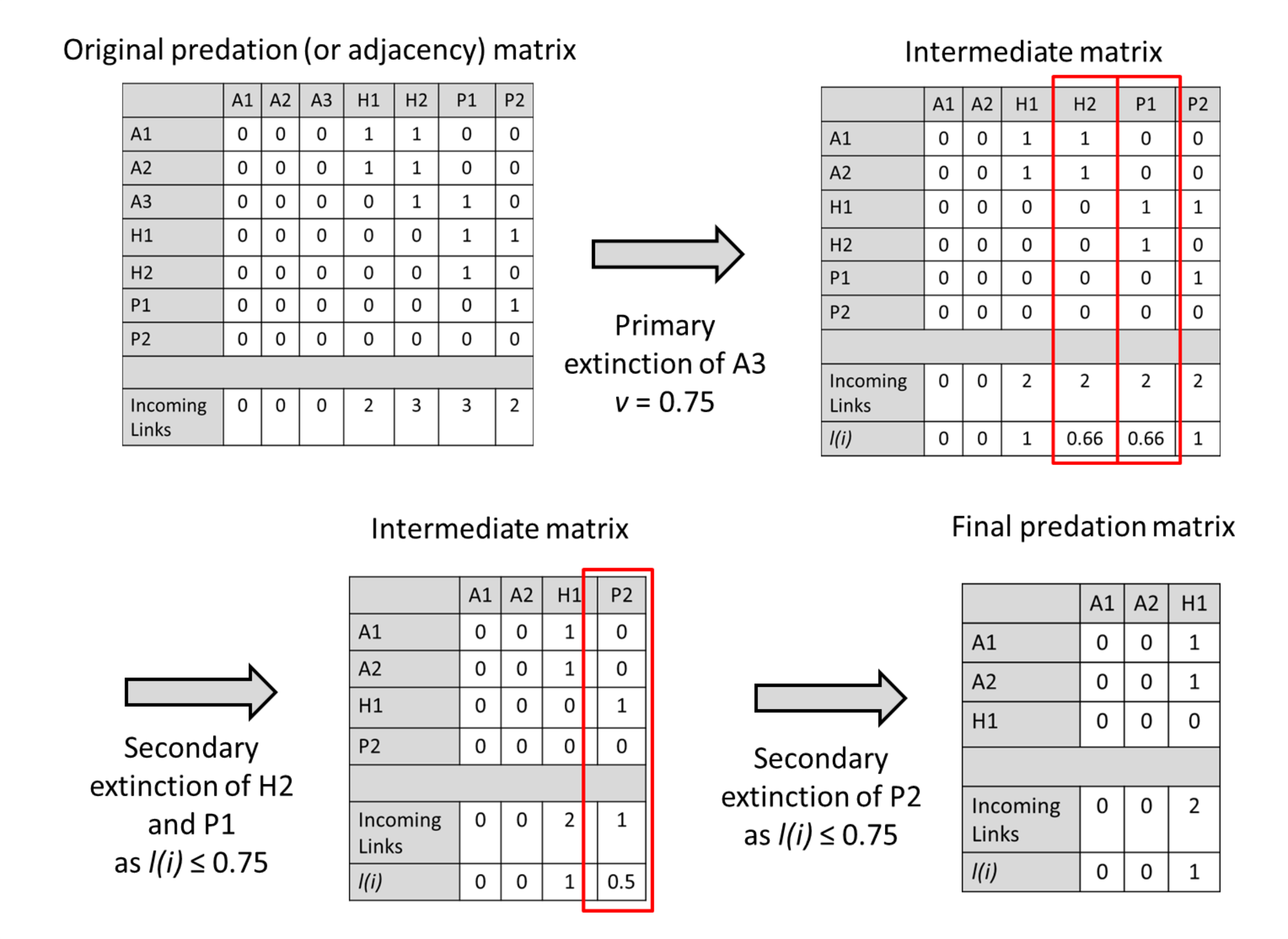

Supplement: Figure S1 — Scheme of the methodology used to detect secondary extinctions after the removal of a particular species (primary loss). In this example, the extinction threshold (v) was 0.75 and H2, P1 and P2 species were secondarily extinct when their fraction of incoming links l(i) was lower than v after the removal of species A3. A1, A2 and A3 represent algae species, H1 and H2 herbivores, and P1 and P2 predators. [file peerj-06-5531-s001.png]
